# Client perceptions of the BreastScreen Australia remote radiology assessment model

**DOI:** 10.1186/s12905-020-01163-7

**Published:** 2021-01-18

**Authors:** Deborah Smith, Karen Johnston, Karen Carlisle, Rebecca Evans, Robyn Preston, Jessamy Beckett, Danielle Geddes, Helen Naess, Melissa Poole, Sarah Larkins

**Affiliations:** 1 College of Medicine and Dentistry, James Cook University, Douglas, QLD 4811 Australia; 2 Anton Breinl Research Centre for Health Systems Strengthening, James Cook University, Douglas, QLD 4811 Australia; 3 School of Health, Medical and Applied Sciences, Central Queensland University, Townsville, QLD 4810 Australia; 4 BreastScreen NSW, PO Box 41, Alexandria, NSW 1435 Australia; 5 BreastScreen NT, PO Box 40596, Casuarina, NT 0811 Australia; 6 BreastScreen Queensland, Level 1, 15 Butterfield St, Herston, QLD 4006 Australia

**Keywords:** Telemedicine, Telehealth, Teleradiology, Breast cancer, Rural and remote, Patient satisfaction, Patient preferences, Models of service delivery, Remote radiology, Breastscreen assessment

## Abstract

**Background:**

Telehealth and teleradiology are increasingly used around the world to facilitate health care provision when the health care provider and clients are separated by distance. The BreastScreen Australia Remote Radiology Assessment Model (RRAM) is an initiative developed to address the challenges of inadequate access to a local radiological workforce in regional Australia. With the growth in telehealth innovations more broadly, the RRAM represents a departure from the traditional onsite model where a radiologist would be co-located with practice staff during assessment clinics. Understanding client satisfaction is an important consideration with new models. This article explores client perceptions of the RRAM including awareness, satisfaction with experiences, confidence in the quality of care being received, and preferences regarding models of service delivery.

**Methods:**

Clients in four BreastScreen services across three Australian states and territories were invited to provide feedback on their experiences of the RRAM. Brief face-to-face interviews based on a survey were conducted at the conclusion of assessment clinic visits. Clients also provided feedback through surveys completed and returned by post, and online.

**Results:**

144 clients completed the survey regarding their experiences of the RRAM. The majority were aged between 50 and 59 years (55/144, 38.2%). Most had attended a BreastScreen service for either screening or assessment on a total of two to five occasions (85/142, 59.9%) in the past. Nearly all women who attended a RRAM clinic expressed satisfaction with their experience (142/143, 99.3%). Clients were aware that the radiologist was working from another location (131/143, 91.6%) and the majority believed there wouldn’t be any difference in the care they received between the RRAM and the onsite model (120/142, 84.5%). Clients generally had no particular preference for either the onsite or RRAM model of service delivery.

**Conclusions:**

Clients’ high satisfaction with their clinic experiences, high confidence in care being received, and the majority having no preference for either the onsite or remote model indicates their acceptance of the RRAM. Client acceptance of the model supports continuation of the RRAM at these sites and expansion. Findings may inform future telehealth innovations where key health care team members are working remotely.

## Background

Worldwide one in four women diagnosed with cancer have breast cancer [1] with 2.09 million cases confirmed in 2018 [2]. In 2019 breast cancer was the most commonly diagnosed cancer in Australian women with 19,535 cases confirmed [3]. Australian women aged between 50 and 74 years are invited every two years to attend a free screening mammogram at their nearest BreastScreen location or outreach service to enhance early detection and treatment [4]. Approximately 54.2% of women in the target age group participate in this screening (1.8 million women in 2016–2017), while only 45.8% of culturally and linguistically diverse women, and 40.7% of Aboriginal and Torres Strait Islander (hereafter Indigenous) women in the target age group are screened. Approximately 11% of women attending their first breast screen and 3.5% of women attending subsequent screens are recalled for further diagnostic investigation at an assessment clinic, following an abnormal screen [1]. At an assessment clinic, further investigation is undertaken of a mammographic abnormality through diagnostic mammography, ultrasound and/or biopsies [4]. BreastScreen Australia has devised National Accreditation Standards (NAS) to promote access to high quality and safe BreastScreen screening and assessment services particularly for women from Indigenous, culturally and linguistically diverse, rural/remote, and lower socioeconomic backgrounds [1].

The lack of health professionals and specialists in rural and remote areas presents challenges for delivering health care to a dispersed population [5, 6]. In 2016, a survey of Australian radiologists found 85.6% were located in a major city [7]. Telehealth, telemedicine and particularly teleradiology, are increasingly used around the world to facilitate health care provision where the health care provider and patients or clients are separated by distance [8]. Teleradiological programmes, where radiological images for diagnosis or consultation are transmitted using information communication technologies, are reported to be in place in 96 countries and regarded as a component of normal working practice in radiology.

The BreastScreen Remote Radiology Assessment Model (RRAM) was developed and piloted by a regional Queensland clinic in response to inadequate access to a local radiological workforce [9]. The RRAM is specific to assessment clinics only; that is, clinics to which clients are recalled after an abnormal screening mammogram. In the onsite model of assessment clinics, a radiologist is co-located with practice staff (medical officers, nurses, medical imaging staff) and may be available to undertake biopsy procedures (although biopsies are generally performed by an appropriately skilled medical officer). The RRAM delivers the same services as the onsite assessment clinic but the radiologist works remotely (in another location) from the BreastScreen team who are present in the clinic. The remote radiologist reviews mammographic images and asynchronous ultrasound images and clips via Picture Archiving and Communication Systems (PACS). Synchronous ultrasound is also available via tele-health technology that facilitates real-time viewing and communication between the remote radiologist and onsite medical officer, and the patient, if desired. Appropriately skilled medical officers perform ultrasound or stereotactic biopsy for clients (when required), with support from radiographic and nursing staff. The remote radiologist discusses each case with the onsite clinical team and determines outcomes to diagnose and advise on client care. In most cases, diagnostic assessment under both models is completed in one day, though in some cases, clients may need to return for biopsy procedures. Discussion of cases involving a biopsy (at a multidisciplinary clinical-pathological meeting) and delivery of results are done similarly in both models. Further details on the RRAM are discussed elsewhere [9].

Whilst many elements of the RRAM may not be immediately apparent to clients, their experiences and expectations of the model are an important measure of the quality of the service. Client satisfaction is an indicator of how well a health service model meets expectations [10]. Despite recommendations in the literature guiding comprehensive evaluations of telehealth interventions [11], few articles have specifically considered client views of teleradiology services. A study conducted in the Netherlands found high levels of client satisfaction regarding the use of an island-based primary care teleradiology service liaising with staff from a mainland hospital [12]. Another study found that 90% of clients surveyed preferred the convenience of radiology being performed locally, which meant wait time was short and they didn’t have to travel, with reading done by a radiologist at the hospital, a distance of 115kms away [13].

James Cook University was contracted by the Australian Government Department of Health to undertake an independent, comprehensive evaluation of the RRAM model. This evaluation was conducted under the guidance of the Governance Committee of the BreastScreen Australia Remote Radiology Assessment Research Project that in turn reports to the BreastScreen Australia National Quality Management Committee. Outcomes relating to safety, quality, implementation and staff acceptability of the RRAM of service delivery are reported elsewhere [9, 14, 15], however this paper will specifically describe clients’ views on the RRAM including client awareness, satisfaction, confidence and their service delivery preferences. Overall, there is a need to better understand client perspectives of teleradiology to ensure client acceptability and ongoing service quality to inform the design of future telehealth services [12]. With the introduction of a novel model of service delivery in the RRAM, it was important to confirm whether or not clients found this model of service delivery acceptable.

## Methodology

Client satisfaction and confidence in the RRAM, awareness and acceptability of the RRAM, and client preferences regarding models of service delivery were evaluated using a client survey. The survey was developed with advice from BreastScreen clinic representatives and a consumer representative (Additional file 1). The survey consisted of Likert-style questions, multiple response questions, open-ended responses, and was implemented in paper format, online using the SurveyMonkey^R^ platform, or through a structured face-to-face interview with a research officer. Demographics collected included age range, Australian Indigenous status, whether a language other than English was spoken at home, postcode and suburb of residence. Clients were also asked if they had experienced telehealth previously. Identifying information was not collected. The client survey was piloted face-to-face with five clients at a RRAM clinic. Minor changes to wording were made to improve the clarity of two questions in response to feedback from clients.

### Participants

A convenience sample of clients at four BreastScreen services across three states and territories in Australia were invited to participate in this component of the evaluation. Two services were in outer regional Australia (Remoteness Area (RA) 3; RA being a measure of relative accessibility and remoteness), one service was located in an inner regional area (RA2), and one service was located in a metropolitan area (RA1) [16]. A research officer attended RRAM clinics at each participating service between June 2018 and July 2019. Three visits were made to clinics at each service, except for the service located in a metropolitan area where only one visit was conducted. Further visits were not undertaken as no other RRAM clinics were conducted during the data collection period.

Two female research officers visited the services to facilitate client input into the evaluation (KJ and DS). Times for a research officer to attend a RRAM clinic for data collection were agreed upon with clinic staff in advance. At the conclusion of their visit to the assessment clinic, staff advised clients of the evaluation and invited them to meet with a researcher. Client participation was entirely voluntary. Clients were provided with an information sheet that outlined: (i) the purpose of the evaluation; (ii) that participation or not would not in any way affect future treatment; and (iii) that no client details were being recorded ensuring confidentiality. Clients providing consent participated in a brief five minute structured interview (based on the survey) with the research officer or chose to complete the survey on an iPad. No-one else was present. Alternatively, clients could take home a paper copy of the survey with a return, postage paid envelope or a project flyer with a link to the survey available online. The first question in all modes confirmed client consent. In the absence of a research officer, staff and volunteers at the participating services distributed surveys (with the project information sheet attached) and return envelopes to clients attending a RRAM clinic. Refusal to meet with a researcher, or the decision to not participate through completion of the paper or online surveys, was not recorded.

### Analysis

Client survey responses collected on paper versions of the survey were manually entered into a Microsoft Excel spreadsheet. Survey responses collected online were retrieved from the SurveyMonkey^R^ platform and added into the dataset. Basic descriptive statistics (frequencies, means and percentages) were used to summarise client preference, satisfaction and perceived quality of care for the RRAM. Open-ended responses were analysed by two researchers using simple content analysis and grouping responses into categories [17]. The research team met to discuss and cross-check emerging categories and associated frequencies. Participants did not provide feedback on findings.

## Results

A total of 144 surveys were completed by clients: 89 surveys were completed verbally with a researcher during visits to the service; 48 surveys were received by mail; and seven (7) were completed online. The majority of surveys were completed in full. Some clients did not provide comments in relation to their responses to the Likert scale questions. Of the 144 surveys, 79 (54.9%) were completed by clients attending a clinic in an RA3 area; 56 (38.9%) were completed by clients attending the RA2 area; and 9 (6.3%) were from clients at the metropolitan service.

### Client demographic characteristics

Demographic characteristics of participating clients are presented in Table 1. Over a third of clients attending the RRAM clinic were aged between 50 and 59 years (55/144, 38.2%) and most had attended a BreastScreen service on two to five occasions in total for either screening or assessment (85/142, 59.9%) in the past. The sample was representative of the wider BreastScreen client population in relation to age and attendance. The majority of clients were non-Indigenous Australians (138/142, 95.8%) with only 2.4% (n = 4) identifying as being Aboriginal and/or Torres Strait Islanders. Just under half of clients (63/143, 44.1%) had travelled 20 kms or less to access the RRAM clinic. However, over a quarter (40/143, 28.0%) had travelled more than 100 km to attend the clinic. Of the clients surveyed the majority (102/140, 72.3%) had not experienced telehealth previously.

**Table 1:** Demographic characteristics of clients participating in the RRAM evaluation

Characteristics	N (%)
Age group (n = 144)	
40–49 years	38 (26.4%)
50–59 years	55 (38.2%)
60–69 years	32 (22.2%)
70–74 years	11 (7.6%)
75 years or older	8 (5.6%)
Indigenous status (n = 142)	
Non-Indigenous Australians	138 (95.8%)
Aboriginal and/or Torres Strait Islanders	4 (2.4%)
Primary language spoken at home (n = 144)	
English	131 (91.0%)
Another language	13 (9.0%)
Total number of visits to a BreastScreen clinic for either screening or assessment (n = 142)	
2–5 times	85 (59.9%)
6–9 times	21 (14.8%)
10 times or more	36 (25.4%)
Distance travelled to access the service (n = 143)	
20kms or less	63 (44.1%)
21–50 km	24 (16.8%)
51–100 km	16 (11.2%)
More than 100 km	40 (28.0%)
Previous telehealth experience (n = 140)	
No	102 (72.9%)
Yes	38 (27.1%)

### Awareness that the radiologist was working from a remote location

The majority of clients (131/143, 91.6%) were aware that the radiologist was working from another location, and not located onsite. Clients reported that they were informed that the radiologist was working remotely, through different methods, and for some at several different times throughout their clinic journey (Table 2). The majority of clients reported that staff had informed them on the morning of the clinic (110/131, 84.0%). Of the 20 clients (15.3%) who reported they were advised by other methods, half of these clients noted it was in written form via letter, information sheet or pamphlet. A handful of clients said they were told by each staff member they saw throughout their journey. One client stated that they were informed

“…on arrival, and [by] each person along the way. Plenty of communication. Helps to feel more relaxed as not everyone has done this.” (Client 69, RA3).

**Table 2:** Methods of communicating the radiologist was working remotely (n = 131)

Methods of communication*	Number and percentage of clients
Staff told me this morning	110 (84.0%)
Staff told me on the phone when making this appointment	35 (26.7%)
When completing the consent form	33 (25.2%)
Other	20 (15.3%)

^*^Clients could choose multiple options

### Satisfaction with clinic experience

All clients but one were ‘extremely satisfied’ or ‘quite satisfied’ with their experience at the RRAM clinic (142/143, 99.3%). Eighty-four percent of clients indicated that they were extremely satisfied with their experience. The majority of clients provided reasons for their high levels of satisfaction. Overwhelmingly, clients’ satisfaction with their RRAM clinic experience appeared to be based on positive interactions with staff members and the way that their care was explained by staff. Interactions were described as friendly, helpful and comforting, and service staff were compassionate and professional:

“The staff were so lovely and caring and explained everything that was happening or going to happen.” (Client 103, RA2).

Other reasons for clients reporting high levels of satisfaction included the efficiency of the staff, the way the clinic was run and being able to have further procedures (such as biopsies) done on the same day. Positive outcomes and perceived high quality of care were also mentioned by some clients. Examples of comments included:

“Seems very efficient, all done in one day, don't have to come back or too long, nice environment, remote radiologist works fine, explained by nurse so you know steps.” (Client 141, RA1)

“Fantastic to be able to consult with multiple doctors on one day and have the various tests done as required.” (Client 110, RA2)

Although the majority of clients reported they were ‘extremely satisfied’ or ‘quite satisfied’ with their clinic experience, a few comments were made that identified opportunities for improvement. These mostly related to the length of time that clients had to wait in the clinic:

“I had expected the appointment might take 30 min, but I was there for almost 4 hours. That was a bit inconvenient, however, I was extremely satisfied that the care was of the highest quality” (Client 95, RA3)

“Very long wait but not their fault the machine broke down. Usually it's fine” (Client 14, RA2).

One client was ‘very dissatisfied’ with their clinic experience but did not make any comments as to why they felt this way.

### Confidence in care

Clients were asked whether they thought the care they received within the RRAM (experienced that day) would be different to the care they might receive under the onsite model with the radiologist physically present at the clinic. The majority of clients did not believe there would be any differences in care between the RRAM and onsite model (120/142, 84.5%). A smaller group of clients felt there would be a difference in care (11/142, 7.7%) and another small group were unsure (11/142, 7.7%).

Clients who felt there would be no difference in care commented that they had not spoken to the radiologist in previous assessments they had attended, and that given the remote radiologist was looking at the same images on the screen then it didn’t matter where they were located. Comments included:

“A doctor met with me, discussed details and will teleconference with radiologist. Having had previous assessments with other private practices I have never personally seen/spoken with a radiologist, so it is irrelevant.” (Client 116, RA3).

“I think it would be the same, because she can see the same screen there and everyone is talking, explaining and clarifying.” (Client 73, RA3)

“Happy to think this is a way of redistributing the resources. Had experience with onsite radiologist before. Happy to talk to medical officer or radiologist.” (Client 143, RA1)

Of the small proportion of clients who thought the care they received would be different, these clients mostly perceived their assessment would be quicker if the radiologist was onsite. A few clients said they would like to have spoken with the radiologist and have the opportunity to ask them questions. A couple of clients felt it was their right to have access to a radiologist onsite from an equity perspective. One client commented: *“I have had a lot to do with the health system. It is really poor that we don't have the same services [in regional area] as they do in [metropolitan city]. There are a lot of people live here and we expect better.” (Client 14, RA2).*

Thirteen clients chose not to answer this question about differences in expected care under the service delivery models by indicating a definite ‘yes’ or ‘no’. However, 11 of these clients provided comments that they ‘didn’t know’ or were unsure if the care they received would be different to the care they might receive with the radiologist physically present at the clinic. Factors they were unsure about included communication processes and use of the technology.

### Preferences for service delivery model

**Fig. 1:**
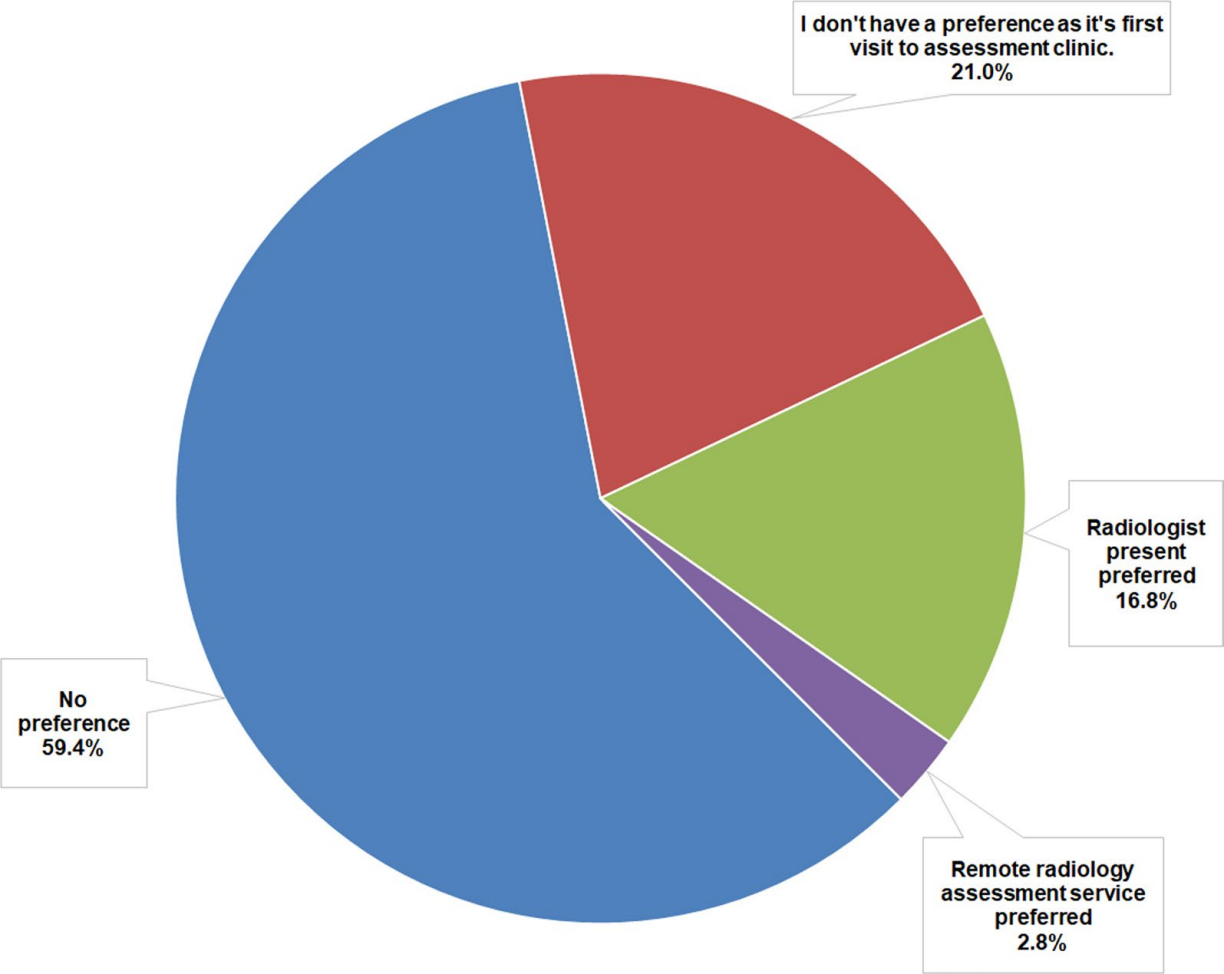
Client preference for service delivery model

The majority of clients (85/143, 59.4%) did not have a preference for any particular assessment service delivery model (Fig. 1). Reasons for choosing ‘no preference’ usually related to a belief that the same diagnostic conclusion would be arrived at under both models of service delivery. Other comments related to clients perceiving they would receive the same level of care under both models, and that the images being viewed were the same no matter where the radiologist was located. Comments included:

“Going on today's experience, I don't see any need for the radiologist to be present. If tossing up [between a] timely response to [having a] radiologist in the room—I would prefer a timely response.” (Client 77, RA3).

“Looking at same images. Doesn't matter where they are.” (Client 140, RA1)

“I think both is good. What has happened today was excellent and if the radiologist was here it would be just as good.” (Client 86, RA3)

A further 30 clients (21.0%) did not have a preference as it was their first visit to an assessment clinic. One client noted that *“…access to experienced staff in the assessment-diagnostic process is most crucial, with onsite or distance-based being secondary in importance.” (Client 88, RA3).*

A preference for the radiologist to be present onsite was reported by 24 clients (16.8%). Some of the reasons for this choice included that the service should be available in a town of a certain size, the right to have equitable access to health care services in rural and regional areas, perceptions that time required in the clinic would be quicker and having the opportunity to ask questions and/or discuss results. Comments from clients about RRAM or onsite service preferences included:

“Around [town] is socio-economically diverse—farmers, lots of mining, lots of money in the community. Lots of FIFO [fly-in, fly-out] money, it should be put into health. We should have better health care.” (Client 14, RA2).

“Maybe quicker, the equipment broke down today.” (Client 17, RA2)

“Would prefer to have a radiologist here to ask questions if needed.” (Client 136, RA1)

Four clients indicated that they preferred the RRAM (2.8%). One client commented on the potential to distribute resources more equitably stating: *“Makes more sense to have a remote radiologist to spread resources equitably.” (Client 143, RA1).*

### Other comments from clients

Just under a third of clients provided final overall comments, an opportunity to provide perspectives that may not have been covered by the survey questions. The majority of comments were positive and summed up their feelings and experiences received in the clinic. Some clients concluded:

“Good that they can do it. Allows specialist diagnosis to be delivered to regional services. Don't have to travel, prompt response and treatment.” (Client 58, RA2)

“I thought it was interesting and we have this technology now and it's one way of providing health care to remote locations.” (Client 81, RA3)

“Seems an innovative idea. Time saving and hope it doesn't put people out of employment here. Expertise here—would prefer local but if not, this is the perfect solution.” (Client 66, RA3)

Only a few final comments noted undesirable experiences and generally related to breakdowns in technology or equipment, and the preference or expectation that a radiologist be onsite.

“Need more reliable hook-up. Not good for ladies to be sitting so long.” (Client 18, RA2)

“Due to population and number of ladies needing assessment, would prefer a radiologist here.” (Client 35, RA3)

## Discussion

The RRAM is a novel model of service delivery for BreastScreen assessment clinics in Australia. For all innovations in health care, evaluation is important for understanding the safety and quality of changed models. The evaluation of the novel RRAM described in this paper reveals the majority of clients were aware that the radiologist was working from a remote location and were satisfied with their RRAM clinic experiences. While the clients in this evaluation were all seen in RRAM clinics, the majority of clients did not expect there would be any differences in care if the radiologist was onsite. The majority of clients did not have a preference for either the onsite or RRAM, although a small proportion of clients reported a preference for a radiologist to be onsite.

There was some concern from service staff regarding client awareness of the RRAM [15], however this proved relatively unfounded with the majority of clients indicating they were aware that the radiologist would be working remotely in their clinic. Although only 25% of women recalled being advised during the phone call to book their assessment visit that the RRAM would be used, it is possible that this was artificially low given the stressful time it is in a women’s life and the influence of recall bias. It was also important in the context of the evaluation to establish that clients were largely aware of the RRAM. Informing clients of new models of care, and measuring their satisfaction ensures the use of technology in health care is with deliberate design [10].

Evidence of an association between client satisfaction and telehealth has been found, although effectiveness in achieving expected outcomes and efficiency varies [12]. Findings in this evaluation support the few published studies relating to clients’ satisfaction of teleradiology which facilitates care close to home [14, 15]. Even though the radiologist was working remotely in RRAM clinics, positive and professional interactions with onsite service staff (e.g. medical officers, nurses, sonographers and radiographers) gave clients confidence in the care they were receiving. Participants largely felt the care they received in the RRAM clinic would be equivalent to that in an onsite clinic. A small proportion of clients expected a difference between RRAM and onsite clinics and these clients felt that onsite clinics may run faster. Supporting this view, service providers participating in other aspects of this evaluation indicated that onsite clinics were faster due to the presence of the radiologist who could often report on imaging immediately, whereas having to communicate with a remote radiologist at scheduled times or as required, tended to slow the assessment process [14]. A few clients also said they would have liked to have spoken with the radiologist and had the opportunity to ask questions. However, a client who had received assessments previously with the radiologist onsite indicated they hadn’t seen or spoken to the radiologist on that occasion either, so perceived there was no difference.

There is evidence that provision of primary care services is associated with more equitable distribution of health and improves overall health across major population sub-groups [18]. It is generally accepted that Australians living in rural and remote areas should have access to appropriate primary health care services and there has been a focus on training and retaining generalist practitioners in these areas, as opposed to specialists [19, 20]. In the context of this evaluation, radiologists with expertise in diagnosing breast cancer is a niche speciality. There are few diagnostic radiologists working outside of major cities in Australia [7] and workforce challenges make it unlikely to access niche speciality services in regional areas.

One client felt that the RRAM was a way of distributing resources and facilitating equitable access to specialists across regional communities. Indeed, telehealth and teleradiology are commonly used in many countries including Australia, to help address the maldistribution of the health workforce and facilitate access to health professionals [8]. Women in low and middle-income countries (LMICs) experience a higher burden and are more likely to die of breast cancer, than those in high-income countries, yet it receives less attention, advocacy and funding in these countries due to competing demands [21]. The World Health Organization (WHO) has a global strategy to improve health workforce issues by 2030 [22] and has initiated mobile health (mHealth) telemedicine programmes to combat some diseases, including some cancers, where there has been a high uptake of smartphones [23]. However, while telemedicine may reduce the acute challenge of geographical maldistribution in some LMIC countries, in the majority of settings access to health workers remains inequitable [22]. In Australia, this evaluation has found that the RRAM successfully makes use of teleradiology to access appropriately qualified radiologists for the provision of timely assessment services for women in rural and remote areas [11, 16, 17].

Women living in rural and remote areas often need to travel to metropolitan and regional centres to access health services. Over a quarter of the clients in this client survey had travelled over 100kms to access the RRAM clinic, and came from smaller rural and remote communities. Some clients had travelled over 1000kms to access the RRAM clinic. International studies found clients preferred to access radiology services locally and minimise the need to travel [12, 13]. More broadly there is evidence that cancer-related telehealth consultations reducing the need for clients to have to travel long distances contributes to satisfaction with services as it is more convenient for them and results in less disturbance to family life [10, 24, 25].

Whilst the majority of clients did not have a particular preference between the onsite or RRAM, equity of access to specialists was an issue raised by some clients who felt that they had the right to be able to access specialist services locally, similar to the level of services available in metropolitan areas. Another study highlighted a perceived barrier to telemedicine was client preference for physical attendances at medical appointments [26], however evaluation of the RRAM found that only a small proportion of clients preferred to have the radiologist onsite. The absence of the radiologist onsite did not appear to have much influence on clients’ confidence in the quality of care they were receiving.

Enabling timely provision of breast cancer assessment services in regional centres reduces anxiety that accompanies the diagnostic pathway. Clients in this evaluation appreciated not having a lengthy wait to attend the assessment clinic, and were satisfied that they could have procedures done on the day by a medical officer. Other components of this evaluation (published elsewhere) found the RRAM delivers safe and high-quality assessment services, with equivalent rates of cancer detection and diagnosis when compared with the onsite model. Timeliness to assessment in the RRAM was significantly improved when compared with the onsite model with 88% of assessment visits commencing within 28 days of clients’ screen dates (benchmark set by the NAS), compared with 62% of assessment visits in the onsite model [15].

Considering the encouraging clinical and timeliness outcomes observed in other facets of this evaluation [15], it was important to confirm clients’ awareness, satisfaction and acceptability of the RRAM. This evaluation indicated that clients: (i) were highly satisfied with their RRAM experiences in BreastScreen assessment clinics; (ii) had high confidence in care received; and (iii) largely had no preference for either the onsite or RRAM. This strongly indicates client acceptance of the RRAM in BreastScreen assessment clinic service delivery. Client acceptability supports continued use of the RRAM and exploration of further expansion. This is particularly important as travel restrictions consequent to the COVID-19 pandemic limit mobility of an interstate radiology workforce, thus stimulating greater uptake of telemedicine alternatives.

### Strengths and limitations

There were clear positive outcomes from clients attending RRAM clinics across all four participating services despite some local contextual differences in delivery. This evaluation provides evidence of client satisfaction with the model, and the use of technology within BreastScreen assessment clinics as a component of a comprehensive evaluation strategy of the RRAM.

This study was conducted in only four clinics operating under the RRAM. A comparison with clients attending clinics operating under the traditional onsite model was not undertaken but may be useful for collecting insights from a comparator group. Client participation rates in the face-to-face interviews, online and postal return of surveys could not be determined. It is possible that there was some non-response bias that could limit the transferability of outcomes to a broader population. In addition, due to the low number of Indigenous clients participating in this evaluation, generalisability of findings to other BreastScreen services providing assessment services for Aboriginal and/or Torres Strait Islander clients and communities may be limited. Broader relevance of these findings to new implementation locations may be influenced by differing local contexts.

## Conclusion

It is important to evaluate innovations in health care delivery, including telehealth models. In this evaluation, clients’ high satisfaction with their participation in the BreastScreen RRAM, high confidence in care, and the majority of clients having no preference for either the onsite or remote model indicates broad acceptance of the RRAM. High satisfaction with the clinic experience appeared to be linked to positive interactions with, and characteristics of, service providers who were described as professional, friendly, helpful, comforting and compassionate, rather than whether or not the radiologist was onsite. The majority of clients did not expect to experience differences in care between the assessment models of service delivery. A small proportion of women reported a preference for a radiologist to be onsite, but this did not impact on their confidence in the quality of care they had received. Client acceptability of the RRAM supports the continuation of the model at these sites and exploration of expansion to further appropriate sites. Findings from this study may inform future telehealth innovations which see key members of the health care team working remotely.

## Supplementary information

** Additional file 1**. Client perceptions survey.

## Data Availability

Raw data are not available for this study due to contractual restrictions. The approved project report may be available upon request from BreastScreen Australia.

## Abbreviations

LMICs: Low and middle-income countries
NAS: National Accreditation Standards
RA: Remoteness Area
RRAM: Remote Radiology Assessment Model
WHO: World Health Organization
