# Supplementary material for: Client perceptions of the BreastScreen Australia remote radiology assessment model
Source: BMC Womens Health. 2021 Jan 18;21:30. doi: 10.1186/s12905-020-01163-7 (PMC7812334; doi:10.1186/s12905-020-01163-7)
Supplement: Supplementary file 1 — Additional file 1. Client perceptions survey. [file 12905_2020_1163_MOESM1_ESM.docx]

**Client perceptions of BreastScreen remote radiology assessments - Survey**

**Is this visit for screening or assessment?**

O Screening *(Disqualification statement: Thank you for considering this survey. No further responses are needed)*

O Assessment (recalled after a screening mammogram). *Please go to Q1.*

**Q1. Do you consent to complete this survey?**

**Q2.**  **How old are you?**

**Q3. Do you identify as Australian Aboriginal or Torres Strait Islander?**

**Q4. When you are at home, do you usually speak a language other than English?**

**Q5a. How satisfied were you with your clinic experience today?**

Please rate your level of satisfaction. Choose one option only.

| 1  Very Dissatisfied | 2  A bit dissatisfied | 3  Neither satisfied or dissatisfied | 4  Quite satisfied | 5  Extremely  satisfied |
| --- | --- | --- | --- | --- |
| O | O | O | O | O |

**Q5b. Please outline a reason for your choice.**

**Q6. Did you know that the clinic today uses a radiologist who is working from another location?**

O No

O Yes

**If yes: How did you know that today’s clinic was a remote radiology clinic?**

(You may choose as many responses as apply)

O staff told me on the phone when making this appointment

O when completing the consent form

O staff told me this morning

O other _________________________________________________

**Q7. Do you think the care you receive today in a *remote radiology assessment service* would be different to the care you might receive with the radiologist physically present here at the clinic?**

O No

O Yes

**Please outline a reason for your choice.**

**Q8a. Do you prefer the radiologist present here or the remote radiology assessment service?**

Please rate your level of preference. Choose one option only.

| Radiologist present preferred | No preference | Remote radiology assessment service preferred |  | I don’t have a preference as this is my first visit to the assessment clinic. |
| --- | --- | --- | --- | --- |
| O | O | O |  | O |

**Q8b. Please outline a reason for your choice.**

**Q9. Is there anything else you would like to add about the remote radiology assessment service?**

**Q10. Have you had any other experience of telehealth?**

**Q11. How many times have you attended (or visited) a BreastScreen clinic altogether (either screening or assessment clinic)?**

**Q12. How far did you travel to access this service?**

**Q13. What is your postcode of residence?**  __________

**Q14. What is your suburb of residence?** _____________
